# Payments for Vaccination

**Published:** 1906-09-29

**Authors:** 


The Hospital

A Journal of

The Medical Sciences and Hospital Administration.

Vol. XL.—No. 1,045. SATURDAY, SEPTEMBER 29, 1906.

### Payments for Vaccination.

The *Times* for August 31 contained a letter "from a correspondent" on the subject of Vaccination, and of the fees now payable to public vaccinators, which has naturally excited a good deal of curiosity alike with regard to the source from which it can have emanated and to the purpose which it was intended to fulfil. The writer commenced by calling attention to the greatly diminished case-mortality by which the small-pox epidemics of the last few years have been attended, and suggested the approach of a time when the disease would be no more formidable than chicken-pox, and when public authorities would naturally hesitate to spend large sums in vaccination for the prevention of what would then be only a temporary and trivial inconvenience. On the strength, apparently, of this golden prospect, he conceived the time to have come when first steps might be taken in the direction of diminishing cost, and he referred to the labours of a "departmental committee" which has already made recommendations for the attainment of the end proposed. We are informed that this committee regard the minimum fees for vaccination specified in the existing order as fairly admitting of reduction, and also that this view seems to be justified by the fact that, whenever a vaccination contract is terminated, there is, at any rate in towns, a superabundance of eager and well-qualified applicants. "Indeed," the writer continues, "it admits of doubt whether inspections of public vaccination at intervals of two years or so can really afford a satisfactory basis for this sort of remuneration, even if it were in principle unobjectionable. There is here an obvious innuendo that the "very capable medical staff" in question has consented, for a great many years, to judge of work and to recommend awards in the absence of any "satisfactory basis" for its conclusions. It would be difficult to formulate a more serious accusation against a body of gentlemen who are not only "capable," but also, in the highest degree, honourable and trustworthy.

It is not the least curious part of the matter that, although the appearance in the *Times* of an unsigned letter "from a correspondent" is usually supposed to indicate something like a general editorial agreement with the views expressed, this does not appear to have been the case in the instance under consideration. The *Times* has always been a strong and consistent supporter of efficient vaccination, and in this respect it has not departed from its traditions. The letter to which we refer was made the subject of a leading article, in which its main positions were disputed or controverted. The statement of the correspondent with regard to the alleged disparity between the fees for vaccination and those paid to district medical officers for operations was disputed; the fee for vaccination being five shillings at the house of the patient, and half-a-crown at the house of the vaccinator, while the scale of fees for operations ranges from one pound to five, according to the severity of the procedure. It was pointed out that the apparent simplicity of vaccination is calculated to obscure both its real character and the vast influence which it may exert over the future safety of the subject, and that it is well to have these emphasised, alike by a reasonably high scale of fees and by a system of inspection calculated to indicate the importance attached by the Government to the proper performance of the operation. It was pointed out that the cost of an epidemic of small-pox is always infinitely more to any locality in which it is suffered to occur than that of the effective vaccination by which its occurrence might have been prevented; and the "correspondent," generally speaking, was left without a leg to stand upon. We cannot but think, however, that his letter should be interpreted as a warning by the profession, and that it may indicate some intention, when the expiring Vaccination Act is superseded by fresh legislation upon the subject, to seek to diminish, by a side issue, the degree of protection now afforded to the public, and to do so at the expense of public vaccinators. The opponents of the operation have been heavily smitten by the experience of Germany, which has conclusively proved alike the efficacy and the harmlessness of vaccination and re-vaccination; and it is by no means unlikely that, hopelessly beaten by the facts of the case, they may yet make some endeavour to undermine the security of the public health under the cover of a pretended desire for economy. It would be well for the Imperial Vaccination League to prepare a careful comparison between the cost of national vaccination and that of the system under which small-pox is permitted to become epidemic in one locality after another, and in which the stable door is never closed until after the steed has been stolen. Since the publication of the *Times* letter referred to, important evidence of the cost of small-pox has been furnished by the issue of the report of the Medical Office of the Local Government Board for 1904-5. It contains an account of an outbreak which affected 1,600 persons in the unvaccinated union of Dewsbury, and by which three districts alone of that union, containing an aggregate population of 46,662 persons, were put to an expense of £20,398, or nearly double the cost of home vaccination for them all!

